# Autoantigen-Harboring Apoptotic Cells Hijack the Coinhibitory Pathway of T Cell Activation

**DOI:** 10.1038/s41598-018-28901-0

**Published:** 2018-07-12

**Authors:** Abraam M. Yakoub, Ralph Schulz, Martina Seiffert, Mark Sadek

**Affiliations:** 1 Department of Molecular and Cellular Physiology, Stanford University School of Medicine, Stanford University, Stanford, CA 94305 USA; 2 Division of Molecular Genetics, German Cancer Research Center (Deutsches Krebsforschungszentrum, DKFZ), Heidelberg, Germany; 3 Faculty of Biosciences, Heidelberg University, Heidelberg, Germany; 4 Department of Pharmaceutical Biotechnology, University of Illinois College of Pharmacy, Chicago, IL 60612 USA; 5 Department of Research and Development, Akorn Pharmaceuticals, Vernon Hills, IL 60061 USA

## Abstract

Apoptosis is an important physiological process in development and disease. Apoptotic cells (ACs) are a major source of self-antigens, but ACs usually evade immune responses. The mechanism by which ACs repress T cell adaptive immune responses is poorly understood. T cell activation is finely regulated by a balance of costimulatory signaling (mediated by the costimulatory receptor CD28 on T cells) and coinhibitory signaling (mediated by the coinhibitory ligands CD80 and PD-L1 and -2 on Antigen-Presenting Cells). Here, we found that ACs specifically upregulated the coinhibitory ligand CD80 on macrophages. Conversely, ACs did not exhibit a robust regulation of the other coinhibitory ligands on macrophages or the costimulatory receptor CD28 on T cells. We show that the robust positive regulation of CD80 by ACs requires phagocytosis of ACs by macrophages. We also demonstrate that CD80 modulation by dead cells is a specific effect of ACs, but not necrotic cells (which stimulate immune responses). These results indicate that ACs modulate the coinhibitory pathway of T cell activation via CD80, and suggest a role for CD80 in suppressing T cell responses by ACs. Understanding a mechanism of regulating adaptive immune responses to ACs, which harbor an abundance of self-antigens, may advance our understanding of mechanisms of regulating autoimmunity and facilitate future therapy development for autoimmune disorders.

## Introduction

Apoptosis is the physiological form of cell death, known to not induce inflammation^1^. ACs are phagocytosed by neighboring cells and by professional phagocytes, such as dendritic cells and macrophages^2^. Phagocytosis of ACs by phagocytes is a complex process^3^.

Accumulating evidence indicates that clearance of ACs actively exerts an anti-inflammatory and immunosuppressive effect. ACs were shown to modulate immunoregulatory cytokine secretion by macrophages toward immunosuppression. They induce the production of immunosuppressive cytokines such as TGF-β and IL-10, but reduce the production of immunostimulatory cytokines as IL-12 and TNF-α^4–6^. In addition to their effects on innate immunity, these cytokines also regulate adaptive immune responses and T cell activation. IL-12, for instance, enhances the differentiation of autoreactive T cells and T cell-mediated autoimmunity^6,7^. IL-10, on the other hand, inhibits the expression of MHC-II and costimulatory molecules required for proper antigen presentation by the antigen-presenting cells (APCs) and activation of T cells, respectively^6^.

With respect to the effect of ACs on adaptive immunity, AC-ingesting dendritic cells were shown to suppress T cell activation and immune responses^8^. Although regulation of cytokine secretion may contribute to the overall effect of ACs on T cells, cytokines alone cannot fully account for the AC effect for various reasons. Firstly, the effects of ACs on production of some cytokines by macrophages can be exerted by only recognition- but not necessarily phagocytosis- of ACs by macrophages^5,9^; however phagocytosis of ACs by dendritic cells was necessary to regulate T cell activation^8,10^. Secondly, the effect of ACs on T cell activation was dominant in presence of lipopolysaccharide (LPS) that upregulates proinflammatory cytokines^8^, suggesting that cytokines are not sufficient alone to account for the effects of ACs. Thus the effect of ACs on adaptive immunity remains to be investigated in depth.

While macrophages can phagocytose ACs *in vivo*^10^ and *in vitro*^11^, regulation of T cell activity by AC-engulfing macrophages and its mechanistic details are unresolved. Previous work suggested that macrophages, similar to dendritic cells, are important APCs for priming T cells and initiating an antigen-specific T cell response. For instance, antigen-presenting macrophages could effectively initiate naïve CD8+ T cell proliferation, activation and differentiation into memory cells *in vivo*^12^. Additionally, macrophages and dendritic cells have distinct localization patterns in the animal body, possibly pointing to the differential relevance of each APC type in different physiological contexts and disease conditions. Macrophages are very abundant at infection or inflammation sites. In some tumors and in nephritis patients, macrophages have distinct tissue infiltration ability and localization pattern than that of dendritic cells^12,13^. Moreover, importantly, macrophages were shown to be essential for the clearance of tumor ACs injected into mice^10^, and ablation of the spleen marginal zone macrophages in mice abrogated the ability of ACs to suppress T cell activation and thus triggered an immune response to ACs^14^. Collectively, these results suggest that the interaction of ACs with macrophages can mediate the effect of ACs, and thus we focused mostly on the AC-mediated regulation of macrophages as the APCs.

Regulation of T cell activation occurs at multiple levels; a particularly important level is during antigen presentation by APCs, which is the initial step leading eventually to differentiation of naïve T cells into certain subsets. Initiation of T cell activation and immune responses requires both antigen (presented on MHC-I or -II) recognition by T cell Receptor (TCR) and costimulatory signals. Costimulatory signals are triggered by binding of costimulatory ligands, most prominently CD86 (B7-2), on APCs to CD28 costimulatory receptor on T cells. Maintaining the balance of T cell activation or inhibition is performed by the coinhibitory signals triggered by binding of CD80 (B7-1) or PD-L (Programmed Death-Ligand)1 (CD274, or B7-H1) or PD-L2 (CD273, or B7-DC) to the coinhibitory receptors CTLA (Cytotoxic T Lymphocyte Antigen)-4 (CD152) or PD (Programmed Death)-1 (CD279), respectively. The relative contribution of both costimulatory and coinhibitory signals determines the activation state of T cells, leading to T cell proliferation or suppression of their activity^15,16^. Although CD80 also can bind to and activate CD28, evidence exists that the function of CD80 is mainly coinhibition. CD80/CTLA-4 binding is of higher affinity than CD80/CD28 binding (*K*_D_ = 0.2 and 4 μM, respectively)^17–19^. CD80/CTLA-4 crystal structure resolution showed that CD80 homodimers bind bivalent CTLA-4 homodimers in a high avidity, unusually stable complex^20,21^ suggested to potentiate their inhibitory signals. Signaling through CTLA-4 is critical for negative regulation of T cell activation and proliferation, as its absence in mice led to severe lymphoproliferation and lymphocytic infiltrates into multiple organs^22^. Therefore, CD80 is essential for suppression of T cell activation.

In this study, we wanted to investigate the mechanism by which ACs engulfed by APCs prevent T cell activation and mounting of an adaptive immune response to them, using macrophages as model APCs. We hypothesized that ACs may prevent activation of T cells by modulating the costimulatory or coinhibitory pathways of T cell activation. We have found that ACs specifically upregulated the coinhibitory ligand CD80 on macrophages and attempted to thoroughly characterize this regulation. Conversely, ACs did not exhibit robust regulation of the other coinhibitory ligands, such as PD-L1 and -2, on macrophages or of the costimulatory receptor CD28 levels on the surface of T cells. These results demonstrate that ACs hijack the coinhibitory pathway via CD80, suggesting a potential mechanism for the suppression of adaptive immune responses by ACs.

## Results

### Effect of ACs on expression of genes that suppress T cell functions

ACs are known to suppress adaptive immune responses and T cell activation. In order to understand how ACs achieve such an effect, we first hypothesized that ACs may be suppressing T cell activation and proliferation by a direct action on regulating genes required for T cell survival, proliferation or activation. To probe for such a direct effect on T cell genes, we arbitrarily tested two important genes that regulate T cell viability or activation, Vascular Endothelial Growth Factor-A (VEGF-A) and Arginase 2 (Arg2). VEGF, aside from its important roles in angiogenesis and tumor growth, has recently drawn much attention for its role in suppressing T cell functions and adaptive immune responses; and VEGF inhibition is currently being investigated as a therapeutic intervention to enhance anti-tumor immunity^23–25^. VEGF secreted from T cells themselves, or from dendritic cells, can act in an autocrine or paracrine manner, respectively, to activate VEGF tyrosine-kinase receptors (VEGFR-1 and -2) on T cells which leads to inhibition of T cell proliferation and inhibition of T cell receptor (TCR)-mediated T cell activation^26^. VEGF and its receptors, VEGFR-1 and -2, are inducibly upregulated upon T cell activation by anti-CD3 or anti-CD28^27,28^. VEGF can also inhibit dendritic cell functions^29–31^.

**Figure 1:**
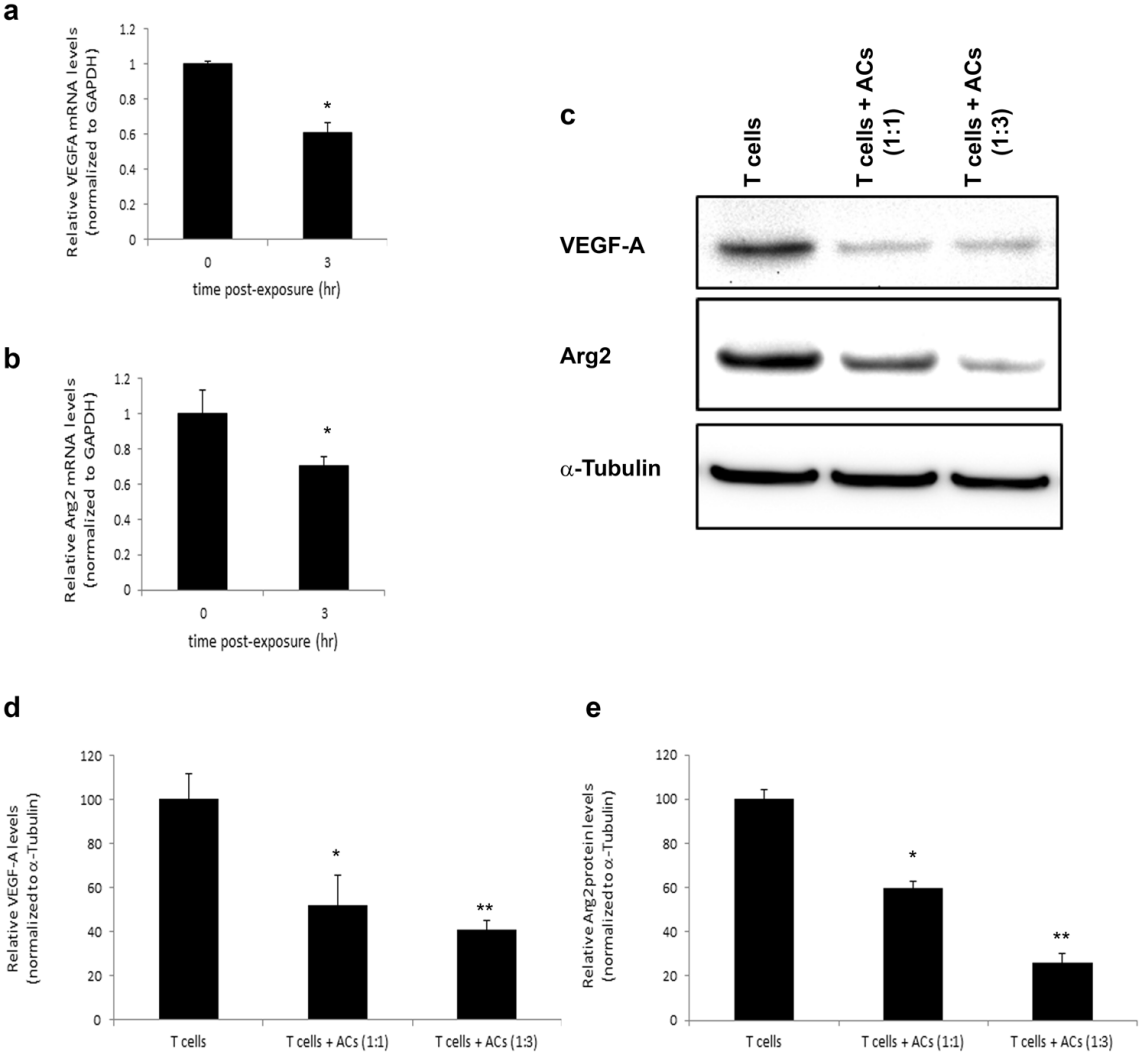
Effect of ACs on T cell expression of select genes important for T cell survival or activation. (**a**,**b**) Jurkat 77 human T cells were exposed to ACs (mouse S49 cells) at a ratio of 10 ACs per T cell for 0 or 3 hours. RNA was then extracted from the T cells and qRT-PCR performed for the indicated genes. Shown are relative gene expression levels for Arg2 or VEGF-A, normalized to GAPDH, at 0 or 3 hours post-exposure to the ACs. (**c**) Jurkat E6-1 human T cells were incubated with apoptotic HeLa cells at the indicated ratios for 6 hours and then immunoblotted for VEGF-A or Arg2; α-tubulin used as a loading control. (**d**,**e**) Quantification of relative protein levels from multiple independent experiments as in (**c**). *p < 0.05, **p < 0.01 (Student’s t-test).

Since ACs regulate the expression of genes associated with innate immune responses, such as TNF (Tumor necrosis factor)-α, at the transcriptional level^4,5^, we first investigated the effect of ACs on transcriptional regulation of genes associated with adaptive immune responses, using quantitative real-time PCR (qRT-PCR) assays. Thus we incubated Jurkat 77 cells (a T cell line derived from human leukemia) with apoptotic S49 cells. After 3 hours we assessed the AC-induced changes in expression of VEGF-A using qRT-PCR. We found that ACs did not enhance the levels of the immunosuppressive VEGF-A, but actually slightly reduced them (Fig. 1a). Thus, we concluded that ACs do not suppress T cell activation via regulating VEGF-A.

**Figure 2:**
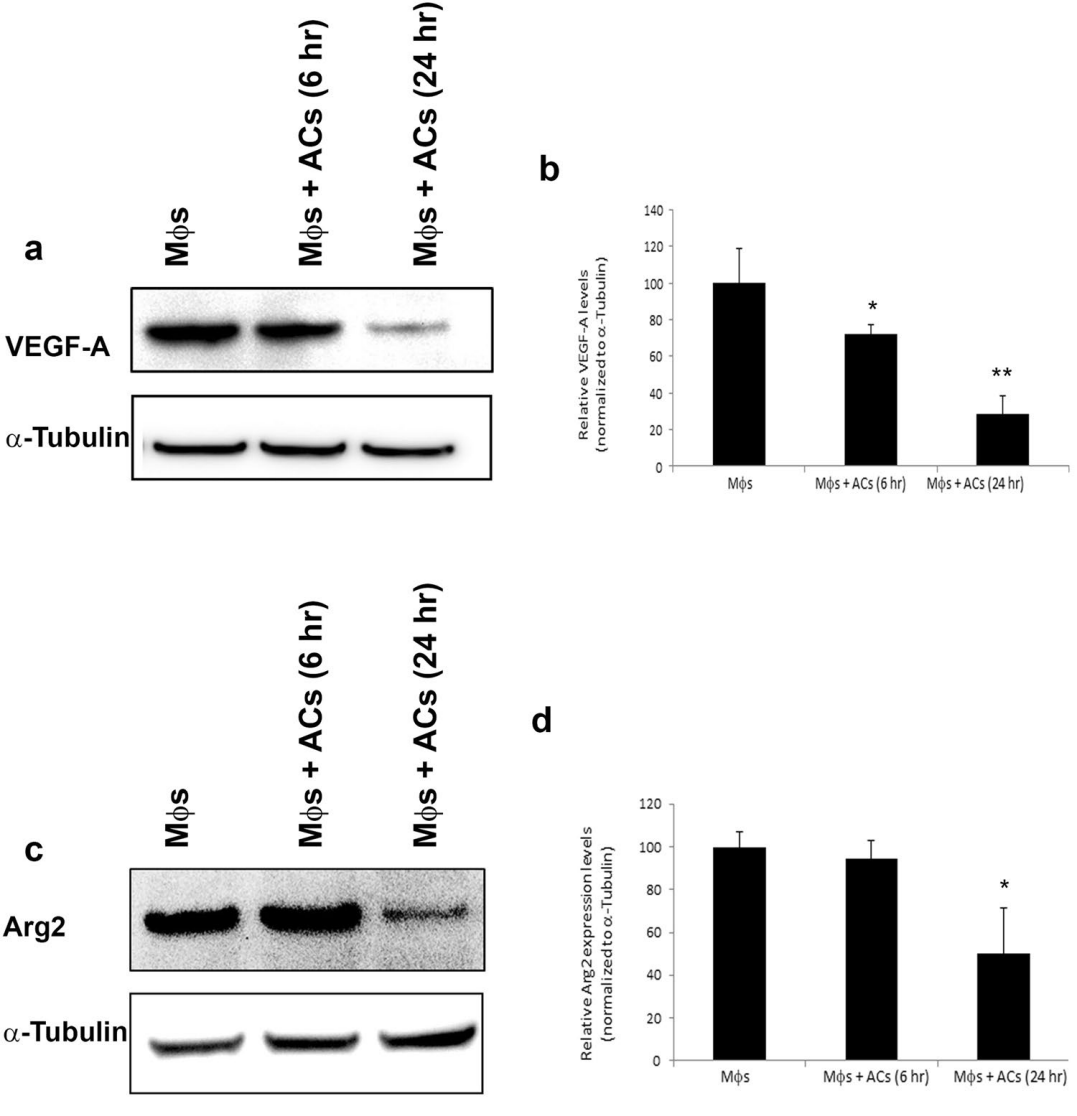
Effect of ACs on VEGF-A and Arg2 expression levels in macrophages. (**a**–**d**). RAW264.7 macrophages (Mϕs) were exposed to apoptotic HeLa cells for 6 or 24 hours and then immunoblotted for VEGF-A (**a**,**b**) or Arg2 (**c**,**d**). (**b**,**d**) Quantification of relative protein levels from multiple independent experiments performed as in (**a**) and (**c**). *p < 0.05, **p < 0.01 (Student’s t-test).

We then tested whether ACs regulate Arg2. Arginase dramatically suppresses T cell proliferation and cytokine synthesis, by depleting arginine in the T cell environment, which leads to CD3ζ chain downregulation without affecting T cell viability per se^32–36^. Moreover, downstream products of the arginine metabolism might generate polyamines and toxic polycationic byproducts that have antiinflammatory properties^37^ or induce apoptosis^38^. Thus, Jurkat cells were exposed to apoptotic S49 cells for 3 hours, and Arg2 expression was tested by qRT-PCR. We found that ACs, similarly to VEGF-A, induced a slight transcriptional downregulation of Arg2, a gene that normally suppresses T cell functions (Fig. 1b). To confirm changes in gene expression levels at the protein level, we performed immunoblotting of T cells exposed to different concentrations of ACs. We found that exposure of T cells to ACs significantly reduced protein levels of VEGF-A and Arg2 (Fig. 1c–e). To test whether ACs modulate VEGF-A or Arg2 production in APCs, which can either have a direct suppressive effect on APC functions or on T cell functions, we measured changes in VEGF-A or Arg2 in RAW264.7 macrophages exposed to ACs. Similarly to T cells, macrophages also showed reduction in VEGF-A levels (Fig. 2a,b) and Arg2 levels (Fig. 2c,d) upon exposure to ACs. Together, we concluded that ACs are unlikely to suppress adaptive immune responses via acting on general genes that regulate survival, or health of T cells or APCs.

### Effect of ACs on the costimulatory receptor CD28

**Figure 3:**
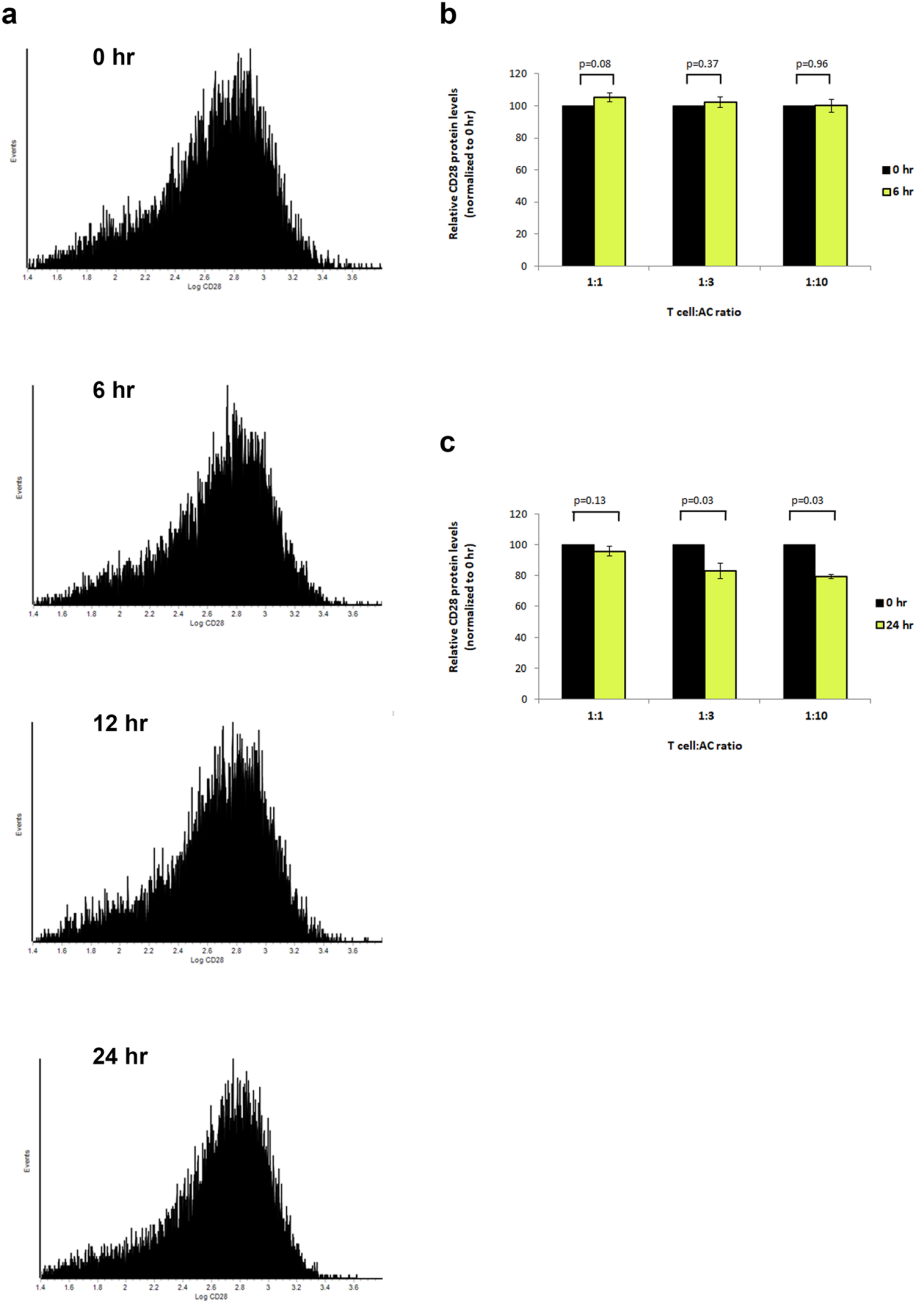
Effect of ACs on CD28 expression on T cells. (**a**–**c**) Jurkat E6-1 T cells were exposed to ACs (apoptotic HeLa cells) for 0, 6, 12 or 24 hours (hr), and CD28 surface expression was determined cytofluorimetrically. (**c**) Changes in CD28 levels on Jurkat E6-1 T cells exposed to ACs at the indicated T cell: AC ratios after 6 (panel b) or 24 (panel c) hours of exposure, normalized to the levels at 0 hours; indicated p-values (Student’s t-test).

Then we reasoned about other mechanisms that regulate T cell functions and activation, and hypothesized that ACs may regulate the costimulatory/coinhibitory signaling that modulates activation of T cells upon encountering APCs. Thus we exposed Jurkat T cells with ACs and measured CD28 levels on the surface of the cells cytofluorimetrically. We could not detect a significant change in CD28 levels on T cells after 6 hours of exposure to ACs (Fig. 3a,b). Even with extended time points (24 hours) and high ratios of AC:macrophage, we could only detect a slight decrease in CD28 protein levels that was barely statistically significant, but we did not observe a robust effect of ACs on CD28 levels (Fig. 3c). These results indicated that ACs do not exhibit robust regulation of CD28.

### Effect of ACs on the coinhibitory ligands on macrophages, PD-L1, PD-L2 and CD80

Given the above result showing no robust regulation of CD28 by ACs and the fact that the effect of ACs on suppressing T cell activation was dominant even in presence of LPS which induces the CD86/costimulatory pathway^39^, we reasoned that ACs may be actively regulating the coinhibitory pathway in order to suppress T cell activation (as coinhibition overrides costimulation). Furthermore, considering that ACs are phagocytosed by macrophages first and possibly regulate T cell activation through macrophages, we decided to investigate whether ACs may regulate the macrophages’ coinhibitory signaling.

Dendritic cells ingesting tumor ACs which activated T cells showed upregulation of the costimulatory ligand CD86 secondary to AC phagocytosis^40,41^. Thus, we found it plausible to propose that T cell-suppressing ACs may upregulate the coinhibitory ligands (CD80, PD-L1 and/or PD-L2). Consistent with our proposal is the fact that these coinhibitory ligand signals are indispensable for suppression of T cell activation^42^. Moreover, lamina propria macrophages that caused suppression of T cell-mediated immune responses in the intestinal mucosa expressed higher levels of coinhibitory ligands than non-immunosuppressive macrophages^43^.

We first considered the possibility that ACs regulate PD-L1 and PD-L2. PD-L1 and -2 are coinhibitory ligands that bind to and activate the coinhibitory receptor PD-1 on T cells in a high affinity binding similar to CD80/CTLA-4 binding^17^, and are both important negative regulators of T cell functions. For example, PD-L1 is upregulated in exhausted CD8+ T cell of lymphocytic choriomeningitis virus (LCMV)-chronically infected mice, and blockade of PD-L1 signaling with anti-PD-L1 antibodies restored CD8+ T cell function and triggered viral clearance^44,45^. PD-L2^−/−^ APCs were also more effective in inducing T cell responses than wild-type APCs, and PD-L2^−/−^ mice showed increased T cell activity over wild-type mice^46^. Double knockout of both PD-L1 and PD-L2, which completely blocks coinhibitory signaling through PD-1, led to stronger T cell activation than single knockout of either gene^47^. This suggested to us that the PD-L1/2 coinhibitory pathway may also be taken advantage of by other immunosuppressive stimuli such as ACs.

**Figure 4:**
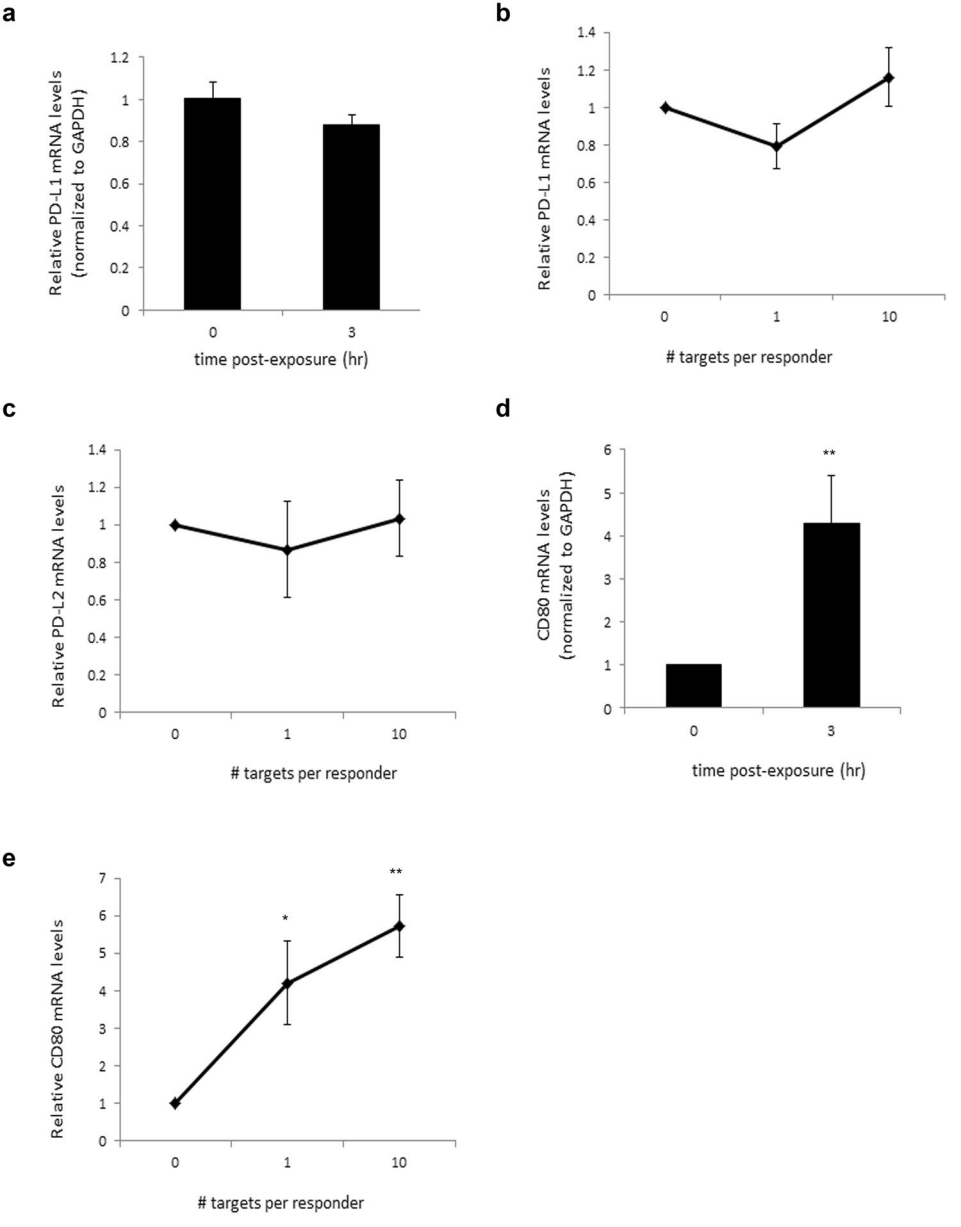
Effect of ACs on macrophage expression of the coinhibiotry ligands. (**a**,**d**) RAW264.7 murine macrophages were exposed to ACs (human Jurkat 77 cells) at a ratio of 10 ACs per macrophage, for 0 or 3 hours. RNA was then extracted from the macrophages and qRT-PCR performed for the indicated genes. Shown are relative gene expression levels for PD-L1 and CD80 at 0 or 3 hours post-exposure to the ACs. (**b**,**c**,**e**) Analysis of the change in expression levels of the coinhibitory ligand genes after 3 hours of exposure to ACs, as a function of the ratio of targets (ACs) per responder (macrophage). After normalization to GAPDH, relative levels at 3 hours post-exposure to ACs were compared to levels at 0 hours and plotted. *p < 0.05, **p < 0.01 (Student’s t-test).

Since PD-L2 is inducibly expressed on dendritic cells and macrophages, we tested whether ACs regulate PD-L1/2 mRNA levels. Thus we incubated RAW264.7 macrophages with apoptotic Jurkat 77 cells, and assessed changes in mRNA expression levels of PD-L1 and -2. We could not detect significant changes in PD-L mRNA levels (Fig. 4a–c) even with increasing the AC:macrophage ratio (Fig. 4b,c). Therefore we concluded that ACs do not significantly regulate PD-L1 and -2.

We then tested the effect of ACs on CD80 expression. RAW264.7 macrophages were exposed to apoptotic Jurkat 77 cells. After 3 hours, we measured changes in CD80 expression levels. Interestingly, we found that ACs significantly enhanced CD80 mRNA levels (Fig. 4d). We then attempted to determine the minimum ratio of AC:macrophage sufficient to produce such an effect. We found that even a ratio of one AC per macrophage was sufficient to significantly induce CD80 expression (Fig. 4e).

### Effect of ACs on CD80 expression in RAW264.7 macrophages and primary murine macrophages

**Figure 5:**
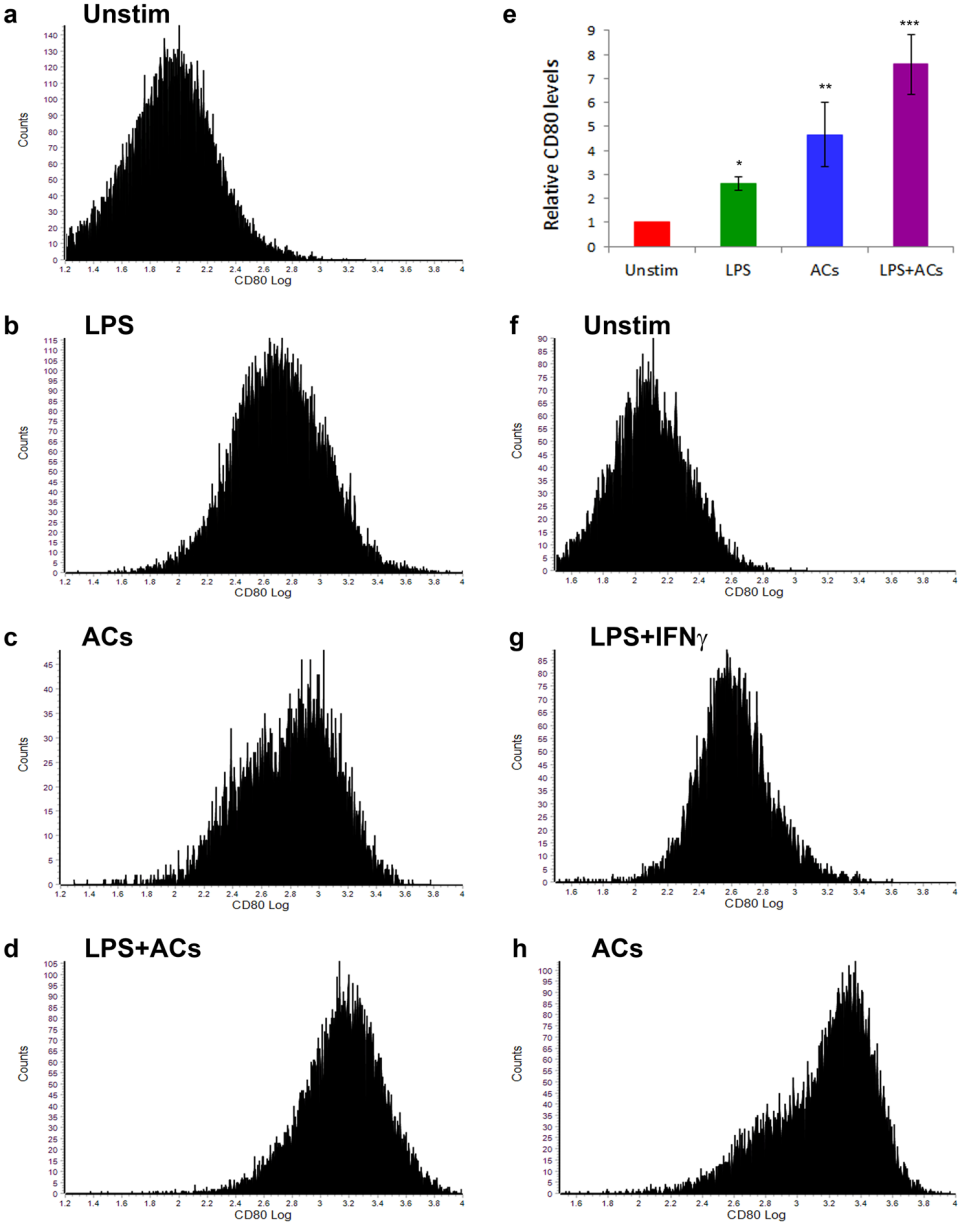
ACs strongly upregulate expression of CD80 on RAW264.7 macrophages and primary murine macrophages. (**a**–**e**) RAW264.7 murine macrophages were exposed to ACs (human Jurkat 77 cells) at a ratio of 10 ACs per macrophage, for 16 hours, and CD80 expression was analyzed using flow cytometry. 10^6^ RAW264.7 cells were plated per well of a 6-well plate 24 hours before ACs or LPS (500 ng/ml) addition (“Unstim” denotes unstimulated cells, exposed to no treatment). 10^7^ ACs (Jurkat77 cells induced to apoptose by 200 ng/mL Actinomycin D treatment for ~12 hours) were added per well. The macrophages were harvested after 16 hours, stained with anti-CD80-FITC and analyzed with flow cytometry. (**e**) The experiment was repeated five independent times, and average CD80 levels were plotted. (**f**–**h**) Primary murine macrophages were exposed to no treatment, LPS + IFNγ or ACs as in (**a**–**e**) and were processed similarly for flow cytometric analysis of CD80 expression as in (**a**–**e**). *p < 0.05, **p < 0.01, ***p < 0.001 (Student’s t-test).

Having seen an effect of ACs on CD80 transcriptional levels, we wanted to confirm the effect of ACs on CD80 protein expression levels on the surface of macrophages. Thus, we incubated RAW264.7 macrophages with ACs, or with a positive control known to induce CD80 expression, lipopolysaccharide (LPS)^48,49^, or the combination of ACs and LPS, and then performed a cytofluorimetric assay for CD80. We found that exposure of macrophages to ACs for 16 hours led to significant upregulation of CD80 expression on macrophages (Fig. 5a–e). Combining ACs with LPS led to an additive effect on CD80 expression (Fig. 5a–e).

To investigate the *in-vivo* relevance of this result, we used primary murine macrophages as model APCs. Thus, primary macrophages were stimulated by exposure to apoptotic cells or a positive control (LPS + IFNγ (interferon γ) combination). Similarly to RAW264.7 cells, primary macrophages also showed a substantial effect of ACs on upregulating CD80 levels on macrophages (Fig. 5f–h). Taken together, these data confirm that ACs induce CD80 expression levels on macrophages.

### In-depth characterization of the effect of ACs on CD80

#### Effect of ACs on CD80 expression on macrophages is specific to ACs, but not necrotic cells (NCs)

**Figure 6:**
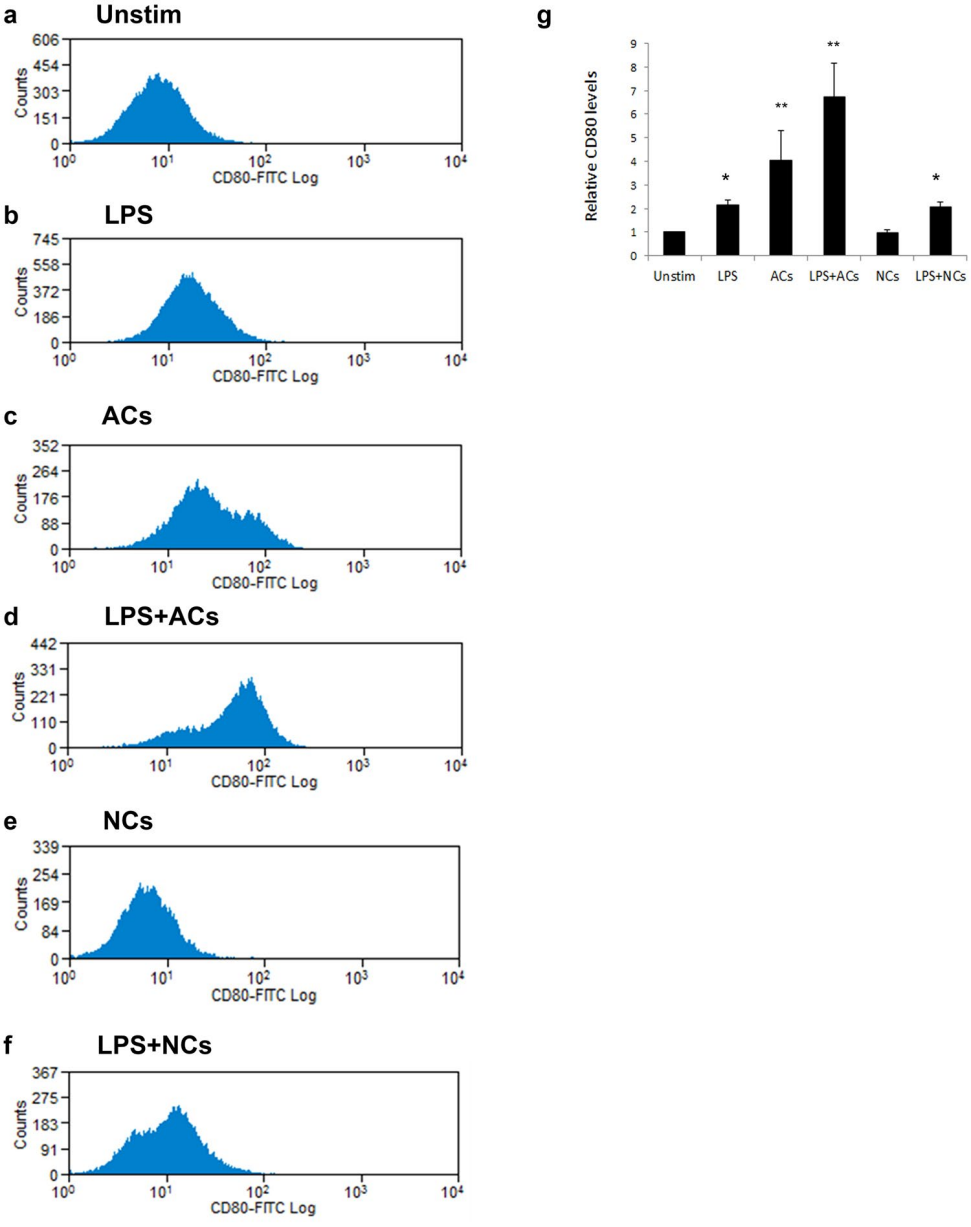
ACs (and not NCs) specifically upregulate expression of CD80 on macrophages. (**a**–**g**) RAW264.7 murine macrophages were exposed to ACs (human Jurkat 77 cells) or NCs at a ratio of 10 ACs per macrophage, for 16 hours, and CD80 expression was analyzed using flow cytometry. 10^6^ RAW264.7 cells were plated per well of a 6-well plate 24 hours before ACs or NCs or LPS (500 ng/ml) addition. (**g**) The experiment was repeated five independent times, and average CD80 levels were plotted. *p < 0.05, **p < 0.01 (Student’s t-test).

Next, we wanted to investigate whether the effect of ACs on CD80 expression is an effect specific to ACs or a nonspecific effect shared by all dead corpses (apoptotic or necrotic). Thus we incubated RAW264.7 macrophages with LPS, dead cells (either apoptotic or necrotic), or a combination of LPS plus dead cells. We then measured macrophages’ CD80 surface expression using cytofluorimetry. While ACs dramatically enhanced CD80 levels, NCs caused no increase in CD80 expression levels (Fig. 6a–g). Thus we concluded that the observed upregulation of CD80 expression on macrophages upon encountering ACs is a specific effect of ACs, suggesting that CD80 upregulation is important for suppressing T cell activation and adaptive immune responses, which is a specific response to ACs not shown by NCs that induce immune responses.

#### Time-course of CD80 upregulation by ACs

**Figure 7:**
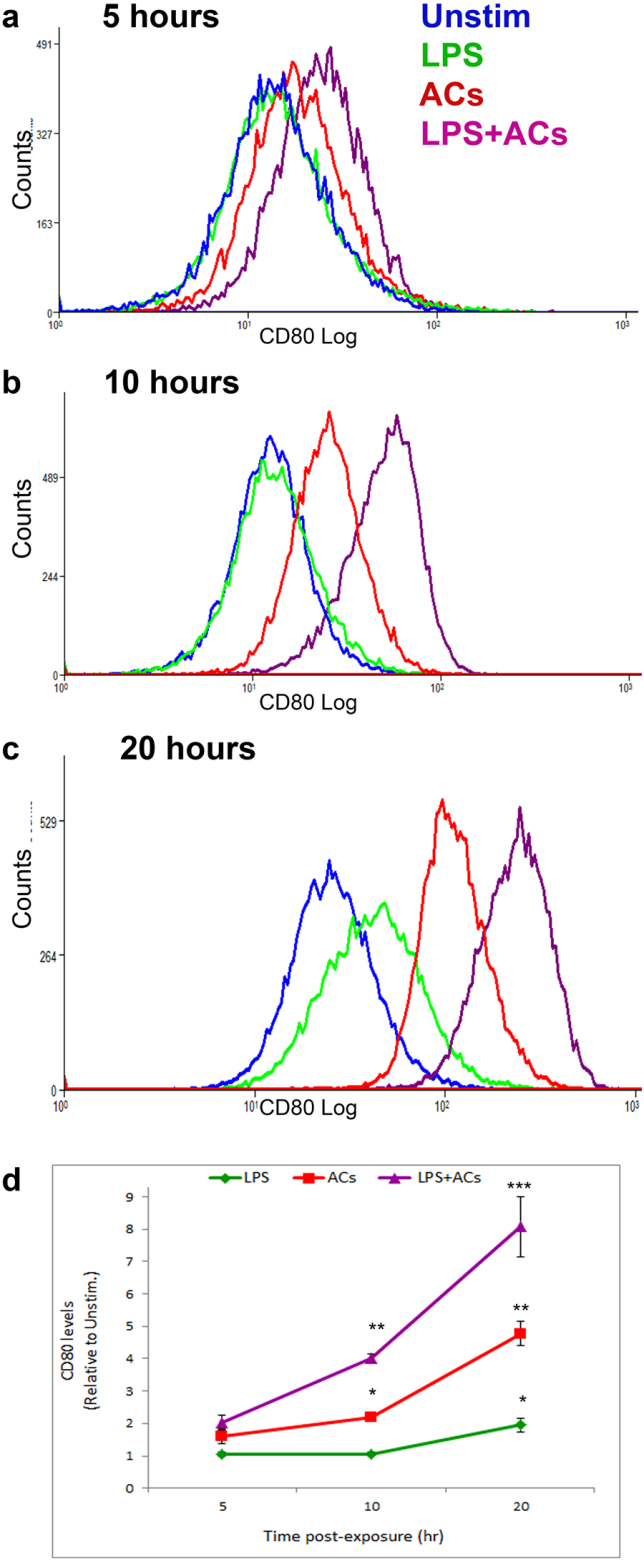
Time-course of AC-mediated upregulation of CD80 on macrophages. (**a**–**d**) RAW264.7 murine macrophages were exposed to ACs (human Jurkat 77 cells) at a ratio of 10 ACs per macrophage, for 5, 10 or 20 hours, and CD80 expression was analyzed using flow cytometry. 10^6^ RAW264.7 cells were plated per well of a 6-well plate 24 hours before ACs or LPS (500 ng/ml) addition. (**d**) Time-course plot of CD80 expression. *p < 0.05, **p < 0.01, ***p < 0.001 (Student’s t-test).

To further characterize the effect of ACs on CD80, we performed a time-course determination of CD80 expression after encountering ACs. RAW264.7 macrophages were incubated with ACs for various durations. At each time point, CD80 expression was assessed using cytofluorimetry. The positive control, LPS, showed no significant effect on CD80 levels after 5 or 10 hours, but showed a modest effect after 20 hours (Fig. 7a–d). Conversely, ACs showed a significant effect on CD80 upregulation as early as 5 hours after incubation with macrophages. Longer durations of exposure to ACs gave further increase in CD80 expression, which always showed an additive effect to LPS when macrophages were exposed to a combination of ACs and LPS (Fig. 7a–d).

#### Concentration and cell-type dependency of the effect of ACs on CD80

**Figure 8:**
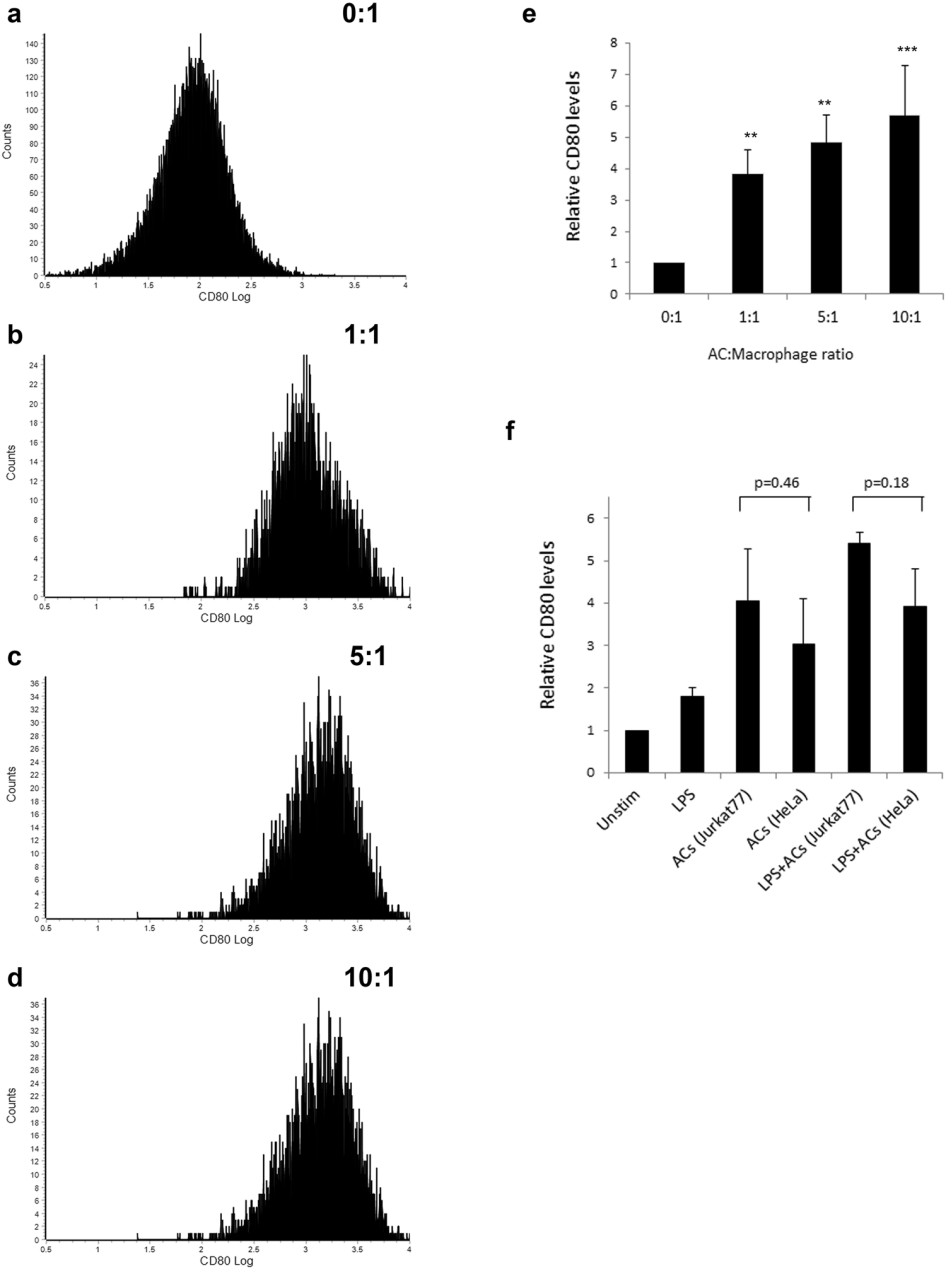
AC concentration, or AC-type, dependency of the effect of ACs on upregulation of CD80 in macrophages. (**a**–**e**) RAW264.7 murine macrophages were exposed to ACs (human Jurkat 77 cells) at the indicated ratios of AC:macrophage for 16 hours, and CD80 expression was analyzed using flow cytometry. (**e**) Quantification of CD80 levels at various AC:macrophage ratios relative to the 0:1 ratio condition (Unstimulated). (**f**) Quantification of CD80 relative levels upon exposure to LPS or the indicated AC types (indicated p-values, Student’s t-test). **p < 0.01, ***p < 0.001 (Student’s t-test).

To further understand the effect of ACs on CD80, we asked whether the concentration of ACs could influence the magnitude or robustness of CD80 upregulation in macrophages. We exposed RAW264.7 macrophages to varying concentrations of ACs, and measured CD80 expression on macrophages using flow cytometry. We found that gradually increasing concentrations of ACs (1, 5 or 10 ACs per macrophage) all upregulated CD80 expression; and the increasing AC concentrations showed subtle, but not statistically significant, differences in CD80 upregulation (Fig. 8a–e). These data indicate that the effect of ACs on CD80 upregulation is independent of the AC concentration. This suggests that any mild production of ACs has a significant effect on upregulating the coinhibitory ligand CD80, and thus possibly suppressing T cell responses and evading autoimmune responses to self-antigens carried on the minimal amount of ACs.

Furthermore, we wanted to investigate if the effect of ACs is dependent on the cell-type of ACs. We thus incubated RAW264.7 macrophages with two different AC types, Jurkat 77 or HeLa cells, and assessed CD80 expression via cytofluorimetry. We found that all AC types tested induced similar effects on upregulating CD80 levels on macrophages (Fig. 8f). This data suggests that upregulation of CD80 by ACs is independent of the cell type of ACs, suggesting that upregulation of CD80 could be a universal mechanism used by all AC types to evade adaptive immune recognition and possibly autoimmune responses to self-antigens carried on/in the ACs.

#### Effect of AC recognition, or phagocytosis, by macrophages on CD80 expression

**Figure 9:**
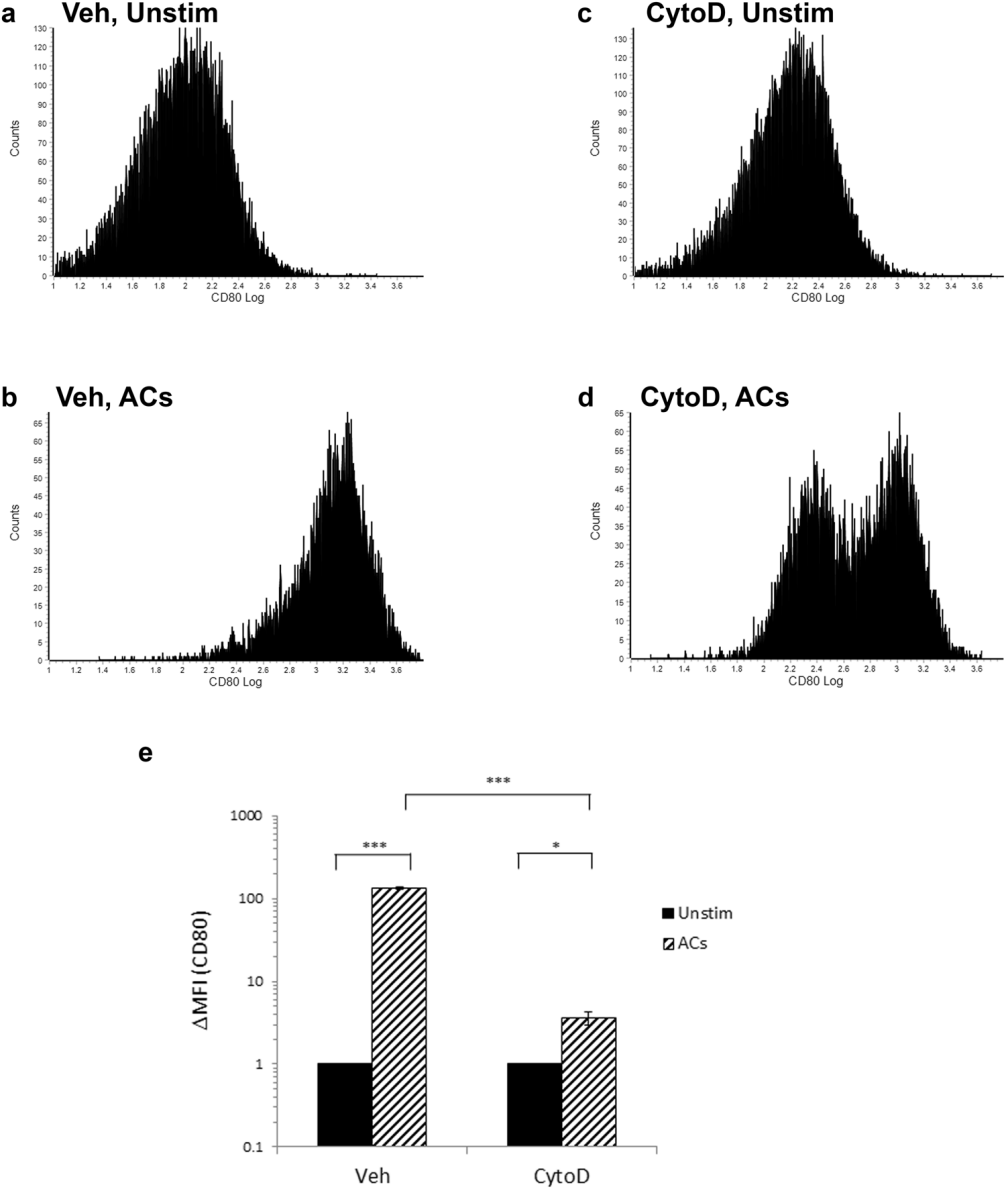
CD80 upregulation by ACs under conditions of functional or blocked phagocytosis. (**a**–**e**) RAW264.7 murine macrophages were exposed to ACs (human Jurkat 77 cells) at a ratio of 10 ACs per macrophage, for 16 hours in presence of vehicle, “Veh” (**a**,**b**) or cytochalasin D, “CytoD” (**c**,**d**), and CD80 expression was analyzed using flow cytometry. The cells were treated with vehicle or 2 μM cytochalasin D starting at 1 hour before addition of the ACs and continuing throughout the incubation period of the macrophages with the ACs. (**e**) Quantification of CD80 relative levels (expressed as ΔMFI (change in mean fluorescence intensity) relative to the Unstimulated control “Unstim”) of the CD80 histogram) upon exposure to ACs in presence of vehicle or cytochalasin D. *p < 0.05, ***p < 0.001 (Student’s t-test).

To fully characterize the regulation of CD80 by ACs, we finally desired to investigate whether upregulation of CD80 required phagocytosis of the ACs by the macrophages or whether only recognition of the ACs by macrophages is sufficient to induce CD80 upregulation. Thus, we tested the effect of ACs on macrophages’ CD80 in presence or absence of the actin polymerization inhibitor, cytochalasin D, which significantly blocks phagocytosis^50,51^. Interestingly, while recognition only of ACs (under blocking of phagocytosis) induced a significant but mild CD80 upregulation, phagocytosis of ACs by macrophages induced a dramatic, much stronger, upregulation of CD80 on macrophages (Fig. 9a–e). This is consistent with the findings in dendritic cells, where phagocytosis of ACs by DC decreased antigen-specific activation of T cell proliferation^8,10^. The requirement of phagocytosis seems to be a specific phenomenon for AC-mediated regulation of adaptive immunity, as AC-mediated regulation of innate immunity and cytokine secretion could be recapitulated by recognition only, but not necessarily phagocytosis, of ACs^5,9^.

## Discussion

In this study we wanted to understand how ACs suppress T cell responses, focusing mostly on the effect of ACs on macrophages. In most cases ACs were shown to dampen T cell responses^14,52–55^. However, sometimes tumor ACs engulfed by dendritic cells^40,41^ or macrophages^10^ could also elicit tumor-specific T cell responses and antitumor immunity. To account for the occasional paradoxical activation of T cells by ACs, two models could be proposed. Firstly, secondary signals form the microenvironment in which AC phagocytosis takes place may work in concert with ACs to favor a certain response. Microenvironments that also contain immunostimulatory stimuli such as TLR (Toll-Like Receptor) ligands or necrotic cells may favor development of autoimmunity, but not tolerance, in response to ACs^56^. Consistently, ACs ingested by dendritic cells, which alone enhanced the development of the immunosuppressive, regulatory T cells (Tregs), enhanced the development of the immunostimulatory Th17 T cells in presence of LPS^57^. However, we focused on uncovering the effects triggered by ACs per se in a ‘neutral’ microenvironment, to understand how AC clearance in physiological contexts mechanistically regulates immune responses to ACs and the AC-associated self-antigens. Secondly, different APC types (dendritic cells or macrophages) in distinct locales possess differential locale-specific activities (activation or inhibition of T cells). Co-ordination of these activities may give rise to a dominant context-specific response. For example, in the intestinal lamina propria there are two types of APCs presenting commensal microbe and dietary antigens to T cells: dendritic cells induce the differentiation of the effector Th17 T cells, whereas macrophages induce the differentiation of Tregs^43^. These Tregs showed increased production of anti-inflammatory cytokines, and decreased production of immunostimulatory cytokines, and were much less proliferative (i.e. became anergic) upon restimulation with the antigen. Since macrophages are much more abundant than dendritic cells in the lamina propria, T cell tolerance is the predominant response in this context. Indeed, that fact further encouraged us to investigate the AC-mediated regulation of T cell activation by macrophages, leading to comprehensive understanding of the immune regulation performed by ACs. Moreover, the idea that ACs may suppress T cell activation through macrophages is plausible, given the fact that macrophage-mediated immunosuppression can reinforce dendritic cell-mediated immunosuppression or predominate the dendritic cell-mediated immunostimulation occasionally observed, macrophages being more abundant than dendritic cells, leading to the overall effect of T cell tolerization to ACs and their associated self-antigens.

Our attempts at unraveling the mechanism by which ACs suppress T cell responses through macrophages demonstrated that ACs take control of the coinhibitory pathway, by inducing a substantial upregulation of the coinhibitory ligand CD80. Importantly, we observed that this effect is specific to AC, while necrotic cells (which stimulate immune responses) failed to produce such an effect on CD80 expression, suggesting that CD80 upregulation may be a potential mechanism- at least in part- used by ACs specifically to suppress T cell activation. Additional mechanisms, however, cannot be completely ruled out, but they are unlikely to play the major role played by CD80 upregulation. For example, the possibility that ACs suppress T cell responses through downregulating the costimulatory ligands such as CD86 is less likely, because CD86 levels in dendritic cells did not change significantly upon uptake of ACs^8,39^. Still, secondary mechanisms, including cytokine-mediated actions, might possibly complement the effect on enhancing coinhibition, as another layer of tightening the regulation of T cells adaptive immune responses to ACs.

We have confirmed the robust effect of ACs in regulating CD80 on macrophages, but how that regulation translates into an actual effect on T cells *in vivo* is an interesting topic for future investigations. Naïve T cells differentiate upon antigen recognition into effector (immunostimulating) T cell subsets such as Th1, Th2 and Th17, or into immunosuppressive Tregs. Dendritic cells ingesting ACs enhanced the development of Tregs, but suppressed the development of the effector Th17 T cells. Although conditioned medium of these dendritic cells, containing their secreted cytokines and soluble factors, could recapitulate such an effect^57^, presence of costimulatory/coinhibitory signaling that takes place during dendritic cell-T cell interaction markedly enhanced Treg proliferation^58^. That indicates that AC-mediated regulation of T cell activation is dependent on direct APC-T cell interaction. Some reports suggest that ACs modulate the maturation of dendritic cells via expression of various surface molecules recognized by T cells which regulate T cell activation and response^8,59^, further highlighting the significance of APC-T cell interaction. Another way in which ACs were thought to regulate T cell responses is through regulating the migration ability of dendritic cells. Dendritic cells migrate upon antigen ingestion, as they mature expressing molecules necessary to prime T cells, to secondary lymphoid tissues such as lymph nodes (LNs) and activate T cells^60,61^. However, such a model of AC action is unlikely, given the fact that AC-ingesting and -noningesting dendritic cells showed comparable migration to the draining LNs^40^. Whether the same is true for macrophages ingesting ACs remains to be determined in *in-vivo* models.

The implications of our results for diseases are abundant, as understanding the mechanisms used by ACs to regulate adaptive immunity will enlighten our understanding of mechanisms that the body uses to regulate immunity in physiological conditions, whose disruption may cause diseases. Firstly, immunosuppression by ACs may serve as a mechanism to prevent auto-immunity to self-antigens carried in ACs; failure of that mechanism might lead to development of auto-immunity. For example, Xia *et al*.^62^ studied the effect of apoptotic β-cell infusion on β-cell antigen-specific CD4+ T cell proliferation and showed that suppression of T cell activation by ACs delayed the onset of diabetes in the autoimmune diabetes-prone (NOD) mice. Thus, our work proposing a mechanism of immunoinhibition by ACs may help facilitate the development of novel effective therapies for autoimmune disorders, such as rheumatoid arthritis, systemic lupus erythematosus and Type I diabetes mellitus. Secondly, immunosuppression exerted by ACs may decrease effectiveness of anticancer chemotherapy, as tumor chemotherapeutic treatment increasingly produces ACs that negatively regulate T cell functions and adaptive immunity. Thus, it is plausible to hypothesize that decreased effectiveness of chemotherapeutic treatments may arise, at least partly, from the progressive inhibition of T cell immune responses by ACs, the by-product of these treatments. Developing mechanisms of functional blockade of the chemotherapy-produced ACs might be a promising key to maintaining effectiveness of chemotherapeutic agents and minimizing their undesirable side effects on the immune system. In fact, some therapies targeting the coinhibitory molecules, which regulate adaptive immune responses by ACs, have been designed^63,64^ and are now in clinical trials^65^.

Overall, our results demonstrate that ACs specifically regulate CD80 levels on macrophages. Inducing coinhibitory signaling through CD80—CTLA-4 binding could either override costimulatory signals, counteracting initiation of T cell activation, or enhance differentiation of immunosuppressive Tregs. Our results highlight the importance of the coinhibitory pathway in suppressing an immune response to ACs, and suggest a potential mechanism of immune regulation that may be used by the body to control reactivity to self-antigens carried by ACs and evade autoimmune responses.

## Methods

### Chemicals

Chemicals were purchased from Sigma Aldrich: Lipopolysaccharide (from *E. coli* O111:B4), Sigma cat # LPS25; Actinomycin D, Sigma cat # A9415; Cytochalasin D, Sigma cat # C8273.

### Antibodies

For CD28 flow cytometry, anti-human CD28-PerCP/Cy5.5 (Biolegend, cat #302922) was used. For immunoblotting, the following antibodies were used: Anti-VEGFA antibody (1:1000) (Abcam, cat # ab46154), Anti-Arg2 antibody (1:1000) (Abcam, cat # 81505), and Anti-α-Tubulin antibody (1:2000) (Sigma Aldrich, cat # T9026). Secondary antibodies used were anti-rabbit IgG (1:5000) (Cell Signaling Technologies, cat # 7074) and anti-mouse IgG (1:5000) (Cell Signaling Technologies, cat # 7076). For cytofluoreimetry of mouse CD80, FITC-conjugated anti-mouse CD80 antibody (clone 16-10A1) purchased from BD Biosciences (cat # 553768) or BD Pharmingen (cat # 560016) was used.

### Cells and cell culture

The following cell lines were used. RAW264.7 is a macrophage cell line derived from an adult BALB/c male mouse. Jurkat 77 and Jurkat E6-1 are T lymphocyte cell lines derived from a human T cell leukemia. HeLa is an epithelial cell line derived from a human cervical epithelium adenocarcinoma. S49 is a murine T lymphocyte cell line derived from lymphoma of BALB/c mouse. Cell lines were obtained from ATCC and were grown at 37 °C in a humidified, 5% (v/v) CO_2_ incubators, as per standard cell-culture procedures. Primary macrophages (from CellBiologics, Cat # C57-6032TF) were isolated as previously described^66^: briefly, mouse peritoneal macrophages were induced by injecting C57BL/6 mice with sterile thioglycollate, and then collected by peritoneal lavage on day 3. Peritoneal macrophages were seeded at a density of 10^6^ cells/well of a 12-well plate ~24 hours before addition of the ACs. After 12 hours, they were observed to have strongly adhered to the plate, then they were washed with PBS and then with media. 12 hours later, cells were re-washed immediately before ACs addition.

### Media

HeLa and S49 cells were cultured in DMEM medium (ThermoFisher) supplemented with 10% fetal bovine serum and penicillin/streptomycin. RAW264.7 and Jurkat cells were cultured in RPMI-1640 (ThermoFisher) also supplemented with 10% fetal bovine serum and penicillin/streptomycin. For induction of primary macrophages, Thioglycollate Medium (Brewer Modified, BD cat # 211716) was used.

### Preparation of Apoptotic Cells

Cells were induced to undergo apoptosis with 200 ng/mL Actinomycin D added in the medium ~12 hours. Apoptosis was verified by flow cytometry per standard procedures.

### Preparation of Necrotic Cells

Cells were induced to undergo necrosis by incubation at 56 °C for 30 minutes (necrosis verified by loss of membrane integrity indicated by trypan blue uptake), immediately before they were added to the macrophages.

### Quantitative Real-Time Reverse-Transcription Polymerase Chain Reaction (qPCR)

RNA was extracted using TRIzol (Invitrogen) as per the manufacturer’s protocol. cDNA was synthesized using High-capacity cDNA Reverse Transcription kit (Applied Biosystems). qPCR was performed using Fast SYBR Green Master Mix and 7500 thermal cycler (Applied Biosystems). All qPCR assays were purchased from Integrated DNA Technologies, IDT (Iowa, US). List of the qPCR assays used:

Human Arg2: Fwd. tggcttgatgaaaaggctct, Rev. cagttcctggttggcaagac

Human VEGFA: Fwd. aaggaggagggcagaatcat, Rev. gggtactcctggaagatgtcc

Human GAPDH: Fwd. atgttcgtcatgggtgtgaa, Rev. gatggcatggactgtggtc

Mouse CD80: Fwd. ttcgtctttcacaagtgtcttca, Rev. tgccagtagattcggtcttca.

Relative gene expression levels were calculated via the 2^−ΔΔ*C*^_T_ method. Gene expression levels were normalized to the housekeeping gene GAPDH which was run in parallel. To rule out any target (ACs) contribution to the RNA of the genes in question, we performed the following precautions and controls: (1) Several, thorough washes of the responders (macrophages or T cells) to clear away all ACs, (2) Inclusion of ACs-only controls to subtract any background contributed by the ACs, and (3) Using targets and responders of different species (mouse S49 targets with human Jurkat 77 responders, and human Jurkat 77 or HeLa targets with mouse RAW264.7 or primary macrophage responders) and then using primers specific for the responder (macrophage or T cell) species only.

### Immunoblotting

On day 0, 5 × 10^5^ RAW264.7 or Jurkat E6-1 cells were plated per well of a 6-well plate. Meanwhile, ACs were prepared by incubating HeLa cells with Actinomycin D (200 ng/mL) for 16 hours. On day 1, ACs were centrifuged and washed 3 times in RPMI-1640 medium. The responder cells were also washed twice with fresh medium before addition of ACs, and then ACs were added to the RAW264.7 cells or Jurkat E6-1 in the wells. At the indicated time points, cells were washed in ice-cold PBS and lysed in RIPA buffer (Sigma Aldrich, cat # R0278) containing 1x Halt protease and phosphatase inhibitor cocktail and 5 mM EDTA (Thermo Fisher Scientific). Protein concentration of samples was measured using the Pierce™ BCA Protein Assay Kit (Thermo Fisher Scientific, cat # 23225). After boiling the samples at 90 °C for 7 minutes, 20 µg protein were loaded on a NuPAGE 4–12% Bis-Tris gel (Invitrogen) and run using NuPAGE MES buffer system (Invitrogen). Proteins were then transferred onto a polyvinylidene difluoride (PVDF) membrane. The membrane was blocked for 90 minutes at room temperature using 5% milk in PBS-T (0.05% Tween 20 in PBS) before being incubated with rabbit anti-Arg2 (1:1000; Abcam, Cat # ab46154), rabbit anti-VEGFA (1:1000; Abcam, cat # ab81505) or mouse anti-α-Tubulin (1:2000; Sigma Aldrich, cat # T9026) antibodies in 5% milk in PBS-T overnight at 4 °C. Secondary antibody staining was performed using an anti-rabbit IgG-HRP (Cell Signaling Technology, cat # 7074) or anti-mouse IgG-HRP (Cell Signaling Technology, cat 7076), respectively, by incubation at a 1:5000 dilution in 5% milk in 0.05% PBS-T for 90 minutes at room temperature. The membrane was finally imaged using Pierce™ ECL Plus Western Blotting Substrate (Thermo Fisher Scientific, cat # 32132) at an Intas ECL Chemostar imager with ChemoStar Imager software (Whole gel images are provided in the online Supplementary Information). Band intensities of immunoblots were quantified using the Plot lane tool for gels of the ImageJ 1.51w software.

### Cytofluorimetry (Flow Cytometry)

On day 0, 10^6^ RAW264.7 cells were plated per well of a 6-well plate. After 12 hours, ACs were prepared by incubating Jurkat 77 cells with Actinomycin D treatment (200 ng/mL) for 12 hours. On day 1, ACs were pelleted and washed 3 times in RPMI-1640 medium. RAW264.7 cells were washed twice with fresh medium before addition of ACs or LPS. ACs in the ratio of 10 ACs per macrophage (unless otherwise indicated) were then added onto the RAW264.7 cells in the wells. For LPS-treated cells, LPS (500 ng/ml) was added to the well. For primary macrophages, the combination of LPS/IFN-γ (100 ng/mL, and 10 u/mL, respectively) was used. 14–16 hours post-treatment (unless otherwise indicated), floating well contents were removed and the adherent RAW264.7 cells were washed three times with 4 mM EDTA/PBS, and then collected by trypsinization. As a control, ACs alone were also used in parallel to control for any possible background coming from the ACs themselves. The cells were washed three times with FACS buffer (1% serum and 0.09% NaN_3_ in Phosphate-Buffered Saline, PBS), and the pellet resuspended in antibody in FACS buffer. We have determined the optimal antibody dilution for best flow cytometry results via empirical antibody-titration experiments; optimal dilution for CD80-FITC was 0.125 μg per 50 μL reaction. Incubation with the antibody took place at 4 °C in darkness for 45 minutes. Afterwards, the cells were washed 3 times with FACS buffer, centrifuged for 4 minutes at 300 g/4 °C, and finally resuspended in 500 μL FACS buffer. The samples were kept on ice in darkness and proceeded to flow cytometry performed on CyAN ADP cytometer (BD). For flow cytometry of CD28, Jurkat E6-1 cells were stained for 30 minutes at 4 °C with anti-CD28-PerCP/Cy5.5 at 1:100 dilution in FACS buffer. To rule out any possible background from the ACs, we (1) co-stained the cells with eBioscience Fixable Viability Dye eFluor 506 (Thermo Fisher Scientific, cat # 65-0866-14) at 1:1000 dilution, and gated the Jurkat cells on the Viability Dye-negative (live cell) population, and (2) performed the 0 time point control (AC added to the Jurkat cells and the cells were immediately fixed and stained) and compared the various time points to the respective 0 time point control. After staining, the cells were washed twice with FACS buffer, fixed in IC Fixation buffer (Thermo Fisher Scientific) and analyzed on a BD LSR Fortessa flow cytometer using the BD FACSDiva software version 8.0.2 (BD).

### Statistical Analyses

Experiments were independently replicated for three to five times. Error bars on the quantification panels represent standard error of the mean (SEM). Statistical significance was determined using Student’s t-test; and a p-value of 0.05 was used as the cutoff for significance.

## Electronic supplementary material

Supplementary Information
