# Supplementary material for: Autoantigen-Harboring Apoptotic Cells Hijack the Coinhibitory Pathway of T Cell Activation
Source: Sci Rep. 2018 Jul 12;8:10533. doi: 10.1038/s41598-018-28901-0 (PMC6043626; doi:10.1038/s41598-018-28901-0)
Supplement: Supplementary file 1 — Supplementary Information [file 41598_2018_28901_MOESM1_ESM.docx]

­­­­­

**Autoantigen-Harboring Apoptotic Cells Hijack the Coinhibitory Pathway of T Cell Activation.**

**Abraam M. Yakoub, Ralph Schulz, Martina Seiffert, Mark Sadek**

**
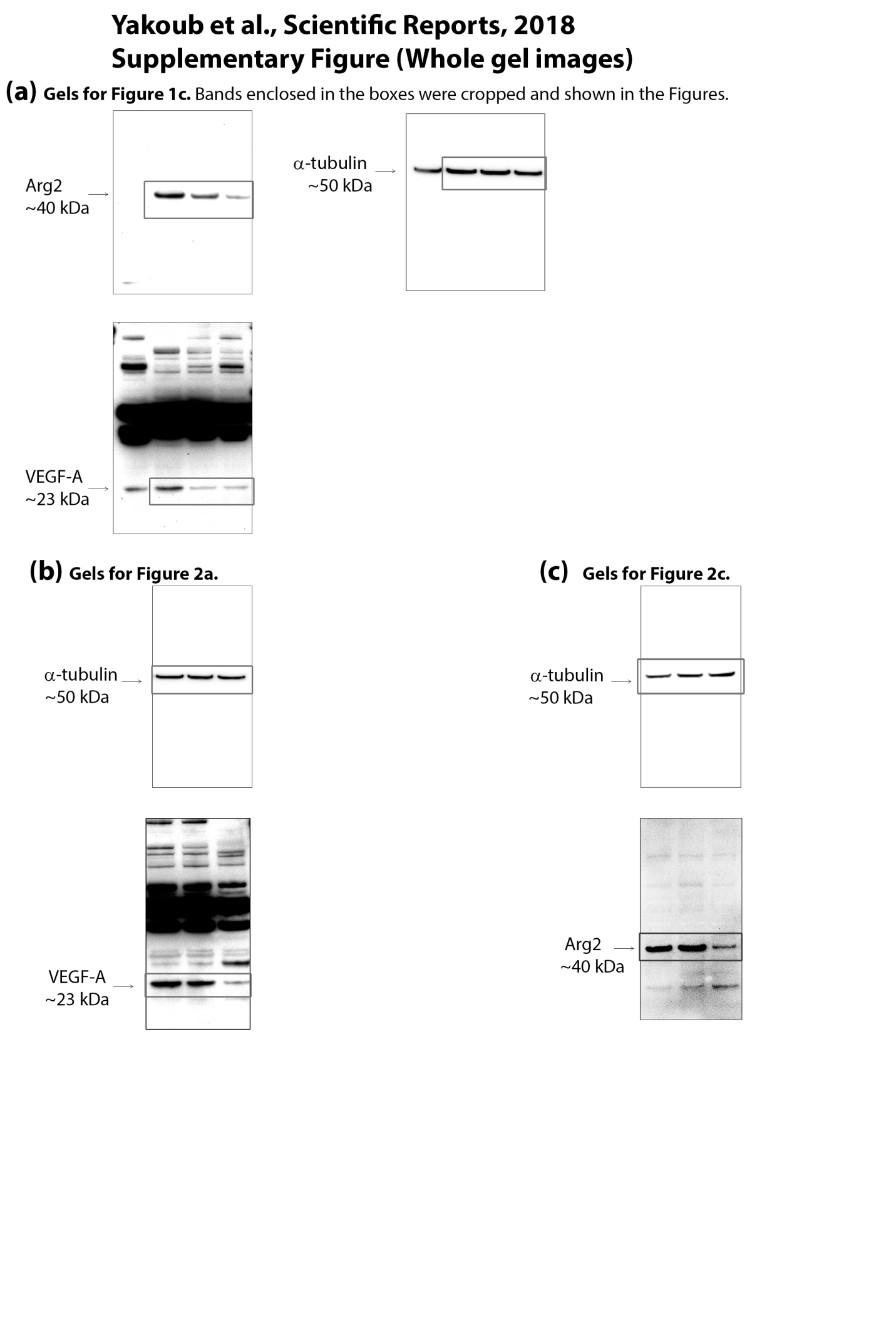
**
